# Trait Depression and Subjective Well-Being: The Chain Mediating Role of Community Feeling and Self-Compassion

**DOI:** 10.3390/bs13060448

**Published:** 2023-05-29

**Authors:** Youming Song, Zijuan Xiao, Lulu Zhang, Wendian Shi

**Affiliations:** 1 Department of Psychology, School of Education, Shanghai Normal University, Shanghai 200234, China; 2 Department of Psychology, School of Education Science, Yan’an University, Yan’an 716000, China

**Keywords:** trait depression, community feeling, self-compassion, subjective well-being

## Abstract

Although subjective well-being has been widely discussed as being one of the important indicators of clinical depression, few studies have explored how it relates to trait depression. In particular, increasing the number of positive experiences has long been a potential goal for depression-related clinical interventions, but the mechanisms by which such interventions work in countering depression have been poorly studied. Grounded in the cognitive theory of depression, the current study aimed to address this specific gap by testing the mediating effects of community feeling and self-compassion between trait depression and subjective well-being. A survey of 783 college students found that trait depression was not only able to directly and negatively predict individual subjective well-being but also indirectly predict individual subjective well-being through the mediating role of community feeling and self-compassion alone and through the chain mediating role of self-compassion from community feeling. These findings reveal the internal mechanisms of trait depression that, to some extent, impede subjective well-being and offer certain guiding significance for the self-regulation of interventions for clinical and non-clinical individuals with trait depression.

## 1. Introduction

Clinical depression has been widely acknowledged as having a particularly destructive effect on individual mental health. In contrast, although trait depression has a higher incidence than clinical depression [1], it has not received the same widespread attention from scholars. Trait depression, as a high-risk factor for clinical depression [2], can directly predict the occurrence of individual major depressive disorder up to two years in advance [3], but how it translates into clinical depression is not yet understood at present. According to the theory of homeostasis [4,5], clinical depression is the loss of subjective well-being due to the failure of the homeostatic defense system; that is, subjective well-being is an indicator of clinical depression. Meanwhile, the cognitive theory of depressionsuggests that the negative bias of depressed individuals’ self-cognition is not only reflected in the relationship between the self and others but also in the internal self [6]. Given that trait depression is a precursor stage of clinical depression [7], its cognitive performance is similar to that of clinical depression to some extent. This means that, as far as the negative bias of self-cognition is concerned, the prediction of trait depression on subjective well-being can be explained through the two perspectives of cognitive bias on the relationships between the self and others and with the internal self.

Trait depression refers to the stable and enduring individual characteristics that are intrinsically associated with the personality dimension of neuroticism [8] and are considered to be distributed widely throughout the general population [9]. Different from clinical depression, trait depression reflects the tendency of an individual, due to their personality traits, to be depressed [10]. This means that, in general, individuals with high-trait depression exhibit depressive symptoms such as pessimism, hopelessness, worthlessness, self-distrust, guilt, and loneliness [11,12] more frequently in their daily lives than other individuals. Trait depression has also been recognized as an important factor in explaining social adjustment disorder in undergraduates [12] and the pathological features of depression relapse [13]. Undergraduates with high trait depression not only show the absence of implicit self-positivity bias [14] but also have a lower level of overall mental health and are more prone to experiencing emotional problems in stressful situations [12,15,16]. It is clear that trait depression is an important risk factor affecting individual mental health.

Subjective well-being is a subjective evaluation of an individual’s satisfaction in all aspects of life, as well as of their levels of positive and negative emotions [17]. According to the theory of homeostasis, people’s subjective well-being is usually at an approximate positive level of 75%, with a normative range of 70 to 80% scale maximum when standardized from 0 to 100 [4]. In contrast, the subjective well-being of individuals with clinical depression will usually be significantly lower than this normal level [5]. As such, subjective well-being is considered to be an indicator of clinical depression. Moreover, the cognitive theory of depression also suggests that, in depressed individuals, cognitive choices of negative biases, such as negative attribution style or negative self-schema [18], can lead to lower emotional levels and to the further accumulation of negative emotional experiences (as a component of subjective well-being) [19]. Therefore, it is unclear how subjective well-being, one of the indicators of clinical depression, is related to trait depression. More importantly, the pursuit of increased positive emotional experiences and subjective well-being in depressed individuals has been the potential target of many clinical interventions for depression, such as interpersonal psychotherapy and cognitive-behavioral therapy. However, less attention has been paid to the theoretical mechanism by which these interventions work. Therefore, an exploration of the relationship between trait depression and subjective well-being can not only help deepen our understanding of the internal mechanism by which trait depression impedes subjective well-being but would also have theoretical significance for the early prevention and intervention of clinical depression.

Despite uncertainty in the mechanisms at play, the relationship between trait depression and subjective well-being is clear. Previous studies have shown that one’s degree of depression is not only significantly correlated with the reduction of subjective well-being [20] but also a significant negative predictor of subjective well-being [21,22]. Since trait depression is a precursor stage of clinical depression [7], the effect of depression on subjective well-being can be extended to the effect of trait depression on subjective well-being. What is not yet clear is the exact manner by which trait depression impedes individual subjective well-being. Returning to the cognitive theory of depression [6,23], depressed individuals have three types of negative biases: first, negative bias towards self-evaluation; that is, depressed individuals tend to self-deprecate and self-condemn; second, negative bias towards the world; that is, depressed individuals think that the world is unfair and treats them harshly; third, negative bias towards the future; that is, depressed individuals tend to deny their own abilities and think that life is without hope. The second type of negative bias reflects the negative cognitive bias of depressed individuals towards their relationship between the self and others, while the first and third types reflect negative cognition and the negation of the depressed individual to the internal self. This means that the negative bias of depressed individuals focuses primarily on the relationship between the self and others and with the internal self. Given that the accumulation of negative emotional experiences caused by the negative bias of depression is an important cause of the decrease in well-being in depressed individuals [19], the relationship between trait depression and subjective well-being can be explained by reduced community feeling and self-compassion.

Community feeling may mediate the relationship between trait depression and subjective well-being. Also known as social interest, community feeling refers to an individual’s main motivation in life and their basis for connecting with others [24], reflecting one’s inner relationship with others and with humanity in general [25]. According to Adler [26], people with high community feelings are often committed to the common good and do not see themselves as having superiority over others. Moreover, they make friends easily and are interested in being useful to others [25]. Previous studies have indicated that hostility and aggression are common in depressed individuals [27], and individuals with depression are more likely to adopt a self-absorbed, passive interpersonal style [28], which leads to a lower level of community feeling. Community feeling, as a necessary condition for a healthy lifestyle [29], as well as an important source of meaningfulness and worth [24,26,30], is not only positively correlated with individual subjective well-being [31], but also an important predictor of individual subjective well-being [25]. Moreover, the results of two meta-analyses [32,33] have suggested that interpersonal psychotherapy (i.e., focusing on improving one’s interpersonal relationships and interpersonal skills) is an effective intervention for alleviating depressive symptoms. In conclusion, lower subjective well-being in trait-depressed individuals is likely to be the result of their lower community feeling, cutting them off from their sources of a sense of meaning and worth.

Self-compassion, meanwhile, may mediate the relationship between trait depression and subjective well-being. Self-compassion is conceptualized as being kind to oneself or having mindful acceptance of one’s suffering when facing inadequacy or failure, as well as accepting that experiencing suffering is common to humanity [34,35,36]. Self-compassion includes three pairs of mutually reinforcing qualities: common humanity—recognizing that life’s difficulties are part of the human experience; self-kindness—being emotionally warm and non-judgmental towards the self in times of difficulty; and mindfulness—being able to acknowledge and observe painful thoughts and feelings [37,38]. As a regulatory strategy, self-compassion can transform negative emotions (such as disgust or shame) into more positive self-referential emotions (such as a sense of kindness or understanding) [34] and thus buffer the effects of low self-related evaluative cognitions on well-being [39,40]. Many studies have also found that self-compassion is not only positively correlated with subjective well-being [41,42,43] but also an important mediator of subjective well-being [41,44]. However, due to their negative bias of self-evaluation, such as in self-deprecation and self-condemnation [6,23], depressed individuals often report low levels of self-compassion [45,46], which may explain their lower subjective well-being. Meanwhile, some intervention studies [47,48] focusing on the connotation of self-compassion have also found that the improvement of self-compassion has a significant positive effect on relieving depressive symptoms. Therefore, the lower subjective well-being of trait depressive individuals may be the result of their lower self-compassion, which is unable to effectively transform negative emotion into positive emotion.

The relationship between community feeling and self-compassion may not be independent. Some researchers believe that the origin of self-compassion can be traced back to an individual’s early attachment experiences [49,50]. More specifically, self-compassion is closely related to maternal warmth and family functioning when one is young [50,51], which develops through being comforted by attachment figures in one’s early life [37] and is the result of the inadequate functioning of the individual threat system and the full development of the comfort system [52]. Coincidentally, community feeling is also formed during one’s first years of life under the influence of an individual’s relationship with their main caretaker (most frequently the mother) and is a relatively stable individual characteristic throughout one’s entire life [24,31]. Therefore, a possible relationship between these two elements could be that the development of self-compassion depends on the establishment of an individual’s community feeling. The reasons for this relationship are two-fold: first, an increased sense of community (referring to the strength of the bond among community members, similar to community feeling) may provide an individual with the necessary guidance and security to develop regulatory skills such as self-compassion [44,53]; second, individuals with high community feeling are more likely to make friends easily and show interest in being useful to others [25], meaning that they are more likely to establish good relationships with others and thus gain further recognition and support. According to attachment theory [54], self-regulation (such as self-compassion) can be developed through positive social relationships, especially those with important others such as parents. That is to say, the decrease in the subjective well-being of trait-depressed individuals is probably due to their lower community feeling impeding the development of self-compassion and thus leads to a decrease in their subjective well-being.

In consideration of the above-mentioned literature, then, this study aimed to explain the relationship between trait depression and subjective well-being from the perspectives of community feeling and self-compassion. The current study hypothesized that the relationship between trait depression and subjective well-being could be explained not only by the indirect effects of community feeling and self-compassion separately but also by the chain indirect effects of self-compassion from community feeling.

## 2. Method

### 2.1. Participants

A total of about 1020 questionnaires were sent out through the Wenjuanxing platform (a professional online questionnaire survey platform; http://www.sojump.com/, accessed on 3 September 2018), and 855 questionnaires were returned completed. After eliminating 72 invalid questionnaires due to obvious repeating patterns in their responses, a total of 783 valid questionnaires were obtained (average age = 19.74 years, *SD* = 1.64, range 15~30), with an effective rate of 91.58%. The sample comprised 346 (44.19%) males and 437 (55.81%) females; 223 (28.48%) freshmen, 321 (41.00%) sophomores, 90 (11.49%) juniors, 138 (17.62%) seniors and above, and 11 (1.40%) unreported grades; 226 (28.86%) only child and 557 (71.14%) non-only child; 535 (68.33%) from rural areas and 248 (31.67%) from urban areas; 323 (41.25%) students majoring in liberal arts, 278 (35.50%) students majoring in engineering, 107 (13.67%) students majoring in art, 68 (8.68%) students majoring in science, 3 (0.38%) students majoring in medicine, and 4 (0.51%) unreported majors.

### 2.2. Measures

#### 2.2.1. Trait Depression Questionnaire

We used the Mandarin trait depression questionnaire, developed by Spielberger [55] and revised by Lei et al. [56], to measure participants’ trait depression. The questionnaire includes 16 items assessing 2 dimensions: dysthymia (comprising 8 items that describe the existence of negative emotions; e.g., “I am unmotivated”) and euthymia (comprising 8 items that describe the absence of positive emotions; e.g., “I enjoy life”). Each item is rated using a 4-point Likert-type scale ranging from 1 (“almost never”) to 4 (“always”). In this sample, the Cronbach’s α coefficient of the total questionnaire was 0.88.

#### 2.2.2. Community Feeling Questionnaire

The community feeling questionnaire, compiled and revised by Kałużna-Wielobób et al. [30] and based on the social interest construct proposed by Adler [24], was used to measure participants’ community feelings. The items in the Chinese version of the questionnaire were translated into Chinese by English majors using the back-translation method, and all items were checked repeatedly and corrected by psychology majors to ensure the accuracy and unambiguous expression of the sentences. The questionnaire includes 46 items measuring 3 dimensions: pro-community orientation (18 items measuring positive tendencies towards public interests and others; e.g., “I would like what I do to serve future generations, regardless of whether they remember my name”); anti-community domination (14 items measuring participants’ sense of competition and dominance over a group; e.g., “Sometimes I disregard people who have not achieved much”); and anti-community isolation (14 items measuring participants’ tendency to feel isolated, anxious, and tense in their interactions with others; e.g., “When I am in different social groups, I often feel alienated”). Items measuring the two dimensions of anti-community domination and anti-community isolation are reverse-scored. Each item is scored using a 6-point Likert scale ranging from 1(“totally disagree”) to 6 (“totally agree”). In this sample, the Cronbach’s α coefficient of the total questionnaire was 0.90.

#### 2.2.3. Self-Compassion Questionnaire

The Mandarin self-compassion questionnaire, as developed by Neff [36] and revised by Gong et al. [57], was used to measure participants’ self-compassion. The questionnaire includes 12 items assessing 3 dimensions: common humanity (comprising 4 items; e.g., “When I fail at something that’s important to me I tend to feel alone in my failure”), self-kindness (comprising 3 items; e.g., “I’m tolerant of my own flaws and inadequacies”), and mindfulness (comprising 5 items; e.g., “When something upsets me I try to keep my emotions in balance”). Items are rated on a 5-point Likert-type scale ranging from 1 (“almost never”) to 5 (“always”), and the higher the total score, the higher the respondent’s level of self-compassion. In this sample, the Cronbach’s α coefficient of the total questionnaire was 0.77.

#### 2.2.4. Subjective Well-Being Questionnaire

The Mandarin version of the subjective well-being questionnaire, as developed by Diener et al. [58] and revised by Yan and Zheng [59], was used to measure participants’ subjective well-being. The scale includes 19 items assessing 3 dimensions: overall life satisfaction (comprising 5 items; e.g., “In most ways my life is close to ideal”), positive emotion (comprising 6 items; e.g., “Happy”), and negative emotion (comprising 8 items; e.g., “Angry”). Items are rated on a 7-point Likert-type scale ranging from 1 (“not true of me at all”) to 7 (“frequently true of me”). In this sample, the Cronbach’s α coefficient of the total questionnaire was 0.78.

### 2.3. Procedure

The study followed a cross-sectional design and was conducted online through the Wenjuanxing platform. Although the use of an online survey can increase potential sampling bias, it does ensure that the survey is voluntary, anonymous, and not restricted by region [60]. Before survey information was made available to potential respondents, study questionnaires were first uploaded to the Wenjuanxing platform, and the link and two-dimensional online survey code were generated accordingly. Using a convenience sampling method, the questionnaire links and two-dimensional codes were randomly distributed to college students at universities in Qinghai, Shaanxi, and Shandong provinces in China. The college student subjects completed the questionnaire online by following a link or scanning the two-dimensional code. Participants were informed that the survey was voluntary and anonymous before beginning to complete the questionnaire online, and each of them was allowed to submit one questionnaire only. The survey was conducted in strict accordance with the Declaration of Helsinki, and all subjects were thanked in writing after completing the questionnaire.

### 2.4. Data Analysis

SPSS 21.0 was used to analyze the Pearson correlations among the variables of interest (i.e., trait depression, community feeling, self-compassion, and subjective well-being) as well as with age, gender, only child status, and home region. The mediating effects were tested using the PROCESS v 3.4 plug-in.

## 3. Results

### 3.1. Common Method Variance Test

To control common method variance, the current study was distributed anonymously, and a commitment to privacy protection was presented to participants before they began the questionnaire. Harman’s single-factor analysis was carried out to assess the severity of data homology errors in this study. The results showed that the data were suitable for factor analysis (KMO = 0.94, Bartlett = 39,166.63, *df* = 5778, *p* < 0.001). The first factor was shown to explain 19.39% of the variation before factor rotation, which is lower than the empirical standard point of 40% [61], indicating that there was no serious common method variance in the measurement.

### 3.2. Descriptive Analysis of Variables of Interest

Pearson correlation analysis of the variables involved in this study showed significant correlations between the main variables of interest (shown in Table 1). Specifically, trait depression was negatively correlated with community feeling, self-compassion, and subjective well-being; community feeling was positively correlated with self-compassion and subjective well-being; and self-compassion was positively correlated with subjective well-being.

### 3.3. Mediation Analysis

We used the PROCESS v 3.4 macro for SPSS to test the mediating effects of community feeling and self-compassion between trait depression and subjective well-being. The PROCESS program is based on the bootstrap procedure, which performs better than the Sobel test and the causal steps approach in terms of statistical power and validity [62]. In testing mediation effects, the criterion for determining whether an indirect effect is significant is whether the 95% bootstrap confidence interval contains 0. More specifically, if the confidence interval for the indirect effect does not contain 0, the indirect effect is significant; otherwise, the indirect effect is not significant [63].

**Figure 1:**
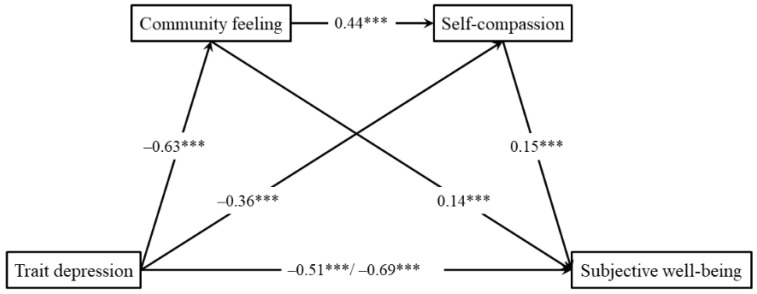
Regression Coefficient of Each Path (*** *p* < 0.001).

In this test, we converted the scores of all variables into *z*-scores and then incorporated them into model 6 in the PROCESS v 3.4 program for analysis. Specific analysis steps are shown in Table 2. Considering that both gender and only child status were related to the main variables we were focusing on (see Table 1), we included both gender and only child status as covariables in the equation. The results showed that the regression coefficients of all paths, including the direct prediction effect of trait depression on subjective well-being, were significant (see Figure 1), indicating that the three sub-mediating models involved in our study were all partially mediating models.

According to the bootstrap estimates (based on 5000 bootstrap samples; see Table 3), both the overall mediating effect and the 3 sub-mediating effects were significant, showing that trait depression not only directly predicted the subjective well-being of individuals but also indirectly predicted the subjective well-being of individuals through the independent mediating effects of community feeling and self-compassion, as well as through the chain mediating effect of community feeling to self-compassion, thus confirming our hypothesis.

## 4. Discussion

Although subjective well-being has been widely discussed as being one of the important indicators of clinical depression [5,64], less attention has been given to the internal mechanism of how it translates from trait depression, and to the theoretical basis of clinical interventions that aim to improve the subjective well-being of depressed individuals. Grounded in the cognitive theory of depression [6,23], the current study explored the relationship between trait depression and subjective well-being from the perspectives of community feeling and self-compassion.

First, we found that trait depression showed significant negative correlations with the community feeling, self-compassion, and subjective well-being, which is largely consistent with the findings of previous studies indicating that depressive symptoms have a significant negative correlation not only with self-compassion [35,38,65,66,67] but also with individual subjective well-being [21]. As for the negative correlation between trait depression and community feeling, this finding is consistent with those of previous studies, which have shown that individuals with a higher level of community feeling are more interested in feeling useful to others [25]. Depressed individuals, in contrast, are more inclined to adopt a self-absorbed, passive interpersonal style [28], showing hostility to others in their daily interpersonal communication [27]. The positive correlations found among community feeling, self-compassion, and subjective well-being were also consistent with the findings of previous studies; for example, Kałużna-Wielobób [25] found a significant positive correlation between community feeling and subjective well-being, while Jeon et al. [41], Phillips et al. [43], and Phillips and Ferguson [42] all found a significant positive correlation between self-compassion and individual subjective well-being. As for the positive correlation between community feeling and self-compassion, this is also consistent with the results of previous studies, which have found that an increased sense of community can provide individuals with the guidance and security necessary to develop regulatory skills such as self-compassion [44].

Second, we found that community feeling played a partial mediating role in the relationship between trait depression and subjective well-being (Path 1), explaining 13.04% of the total effect and 50.00% of the total indirect effect. Community feeling, as one’s inner relationship with others and with humanity as a whole [24], reflects one’s attitude toward and evaluation of the relationship between themself and the rest of humanity. Previous studies have shown that individuals with depression are more likely to adopt a self-absorbed, passive interpersonal style [28], which means that their level of community feelings is lower. When individuals have lower community feeling, it becomes challenging for them to sustain a healthy lifestyle, and they may experience a lack of value and meaning in their lives. Community feeling is a necessary condition for the promotion of a healthy lifestyle [29] and is also a crucial source of meaning and value in one’s life [24,26,30]. Furthermore, community feeling is not only positively correlated with an individual’s subjective well-being [31] but is also an important predictor of it [25]. Therefore, when individuals have higher trait depression, they will generally also have a lower community feeling, which makes it difficult for them to find meaning and value in life, thus resulting in them experiencing low subjective well-being.

Third, we found that self-compassion also played a partial mediating role in the relationship between trait depression and subjective well-being (Path 2), explaining 7.25% of the total effect and 27.78% of the total indirect effect. Self-compassion is regarded as an affect regulation strategy in which individuals treat themselves kindly, consciously accept their own suffering when facing deficiencies or failures, and believe that experiencing suffering is a universal human phenomenon [34,35]. Our findings support the understanding of self-compassion as being not only a buffer against the influence of low self-related evaluative cognition in the subjective well-being process [39,40] but also an important mediator of one’s subjective well-being [41]. However, due to the negative bias of self-evaluation in depressed individuals, coming out as self-deprecation or self-condemnation, for example [6,23], numerous studies have confirmed that depressed individuals have lower levels of self-compassion [45,46] which means that these depressed individuals are less likely to treat themselves kindly in the face of their own inadequacies or failures, and thus experience lower levels of subjective well-being. Meanwhile, self-compassion can be seen as acting as an emotional regulation mechanism in cognitive function and transforming negative emotions into more positive self-referential emotions [34], thereby eliminating negative emotions accumulated by individuals throughout daily life. This is consistent with the theorized connection between depression and abnormal emotional regulation [68], that is the belief that depressed individuals have emotional disorders which manifest largely through the reduced use of adaptive strategies (such as cognitive reappraisal) and the increased use of non-adaptive strategies (such as expressive suppression). In other words, due to their low level of self-compassion, individuals with a high level of trait depression are unable to effectively regulate their own negative emotions and interpret their own failures and deficiencies with a self-critical cognitive perspective [45] which ultimately leads to a decline in their subjective well-being.

Finally, we found that community feeling and self-compassion played a chain mediating role in the relationship between trait depression and subjective well-being (Path 3), explaining 5.80% of the total effect and 22.22% of the total indirect effect. Specifically, the negative effect of trait depression on subjective well-being appears to be realized through the reduction of one’s community feeling, which thereby reduces self-compassion. Attachment theory suggests that positive social relationships precede the development of emotional regulation skills [54], while self-compassion, as an affect regulation strategy, can convert negative emotions into more positive self-referential emotions [34]. Low community feeling in trait-depressed individuals causes them to be less likely to relate to the important people around them when they are young, which further inhibits their development of self-compassion and ultimately leads to a decline in their subjective well-being. In other words, the low community feeling of trait-depressed individuals deprives them of the opportunity to further develop self-compassion, which makes it difficult for them to transform and dispel the accumulated negative emotions they experience as a part of daily life in a timely and effective manner, thus leading to a decline in their subjective well-being.

It is important to note that, although our findings have revealed the intrinsic mechanisms of how trait depression to some extent impedes subjective well-being, 73.91% of the total effect remains unclarified by our study, which means that in addition to the direct effect of trait depression on subjective well-being, there may be other mediating mechanisms at play. Previous studies have found that reduced mental resilience is associated with depression [69,70], while subjective well-being can also be explained by mental resilience [71,72]. Therefore, mental resilience may also be a key internal mechanism by which trait depression impedes subjective well-being. Similar psychological variables include cognitive reappraisal [68], self-evaluation [73,74], and self-esteem [75,76], among others. Therefore, the underlying mechanism of trait depression’s impediment to subjective well-being still merits further exploration.

## 5. Implications and Limitations

The present study examined the relationship between trait depression and subjective well-being, as well as the mediating role of community feeling and self-compassion. Its practical significance lies in the fact that both the self-regulation of individuals with trait depression and clinical interventions should root themselves in two aspects, namely self-compassion and, particularly, community feeling. Indeed, the Adlerian Adventure Play Therapy Program has been shown to significantly improve the community feeling of preadolescent children [29], while the Mindful Self-Compassion Program [77] has been proven to significantly improve individuals’ level of self-compassion. Therefore, clinical workers are encouraged to use tools such as the Adlerian Adventure Play Therapy Program and the Self-Compassion Program to intervene via the community feeling and self-compassion pathways, respectively, in clinical and subclinical depressed individuals. More importantly, in view of the positive value of community feeling in promoting self-compassion, addressing depression through interpersonal psychotherapy with a focus specifically on community feeling may be an effective way to further improve the intervention effect, especially long-term.

There are, of course, some limitations in the current study. First, the method adopted in this study was a cross-sectional design, which cannot fully reveal causal relationships between research variables. Second, our research subjects were all college students, meaning that the extent to which the conclusions can be extended to other populations is unknown. Third, the data of this study was obtained through online surveys, which may lead to certain sampling biases. Fourth, the community feeling questionnaire used in this study has not been revised specifically in the context of Chinese culture, so the validity of the conclusions of this study must be further examined through subsequent studies. Fifth, the impeding mechanism of trait depression on subjective well-being (or the mechanism of depression-related clinical intervention) is not comprehensive, and there may also be other intermediary mechanisms at play, such as mental resilience, cognitive reappraisal, self-evaluation, and self-esteem. Finally, although trait depression reflects an individual’s tendency toward depression, it is different from other true personality traits in the representation of personality structure. As such, the theoretical model obtained in this study requires further validation using personality models associated with depression, such as neuroticism and extraversion [78]. Future studies should, therefore, further verify the conclusions of our study by developing alternative research designs, broadening the research sample, revising the Chinese version of the community feeling questionnaire, enriching the intermediary mechanism, and increasing the evidence related to depression regarding personality models in order to make the research results more reliable and ecologically valid.

## 6. Conclusions

The relationship between trait depression and subjective well-being is not only mediated by community feeling and self-compassion alone but also by the chain mediation of community feeling to self-compassion.

## Figures and Tables

**Table 1:** Bivariate Correlations Between Variables of Interest (*n* = 783).

	1	2	3	4	5	6	7	8
Age	1.00							
2. Gender ^a^	0.24 ***	1.00						
3. Only child status ^b^	0.12 ***	0.18 ***	1.00					
4. Home region ^c^	0.08 *	0.04	0.38 ***	1.00				
5. Trait depression	−0.002	0.10 **	0.06	−0.02	1.00			
6. Community feeling	0.06	−0.10 **	−0.06	0.07 *	−0.63 ***	1.00		
7. Self-compassion	0.01	−0.15 ***	−0.09 *	−0.002	−0.65 ***	0.68 ***	1.00	
8. Subjective well-being	−0.02	−0.10 **	−0.03	0.003	−0.70 ***	0.57 ***	0.57 ***	1.00
*M*	19.74	0.56	0.71	0.68	34.13	179.33	40.10	22.20
*SD*	1.64	0.50	0.45	0.47	6.67	23.84	6.52	15.70

Notes: ^a^ male = 0, female = 1; ^b^ 0 = yes, 1 = no; ^c^ 0 = urban area, 1 = rural area; * *p* < 0.05, ** *p* < 0.01, *** *p* < 0.001.

**Table 2:** Results of Mediating Effect Test of Community Feeling and Self-Compassion (*n* = 783).

Regression Equation		Significance of Regression Coefficient						Overall Fit Index			
Result Variable	Predictors	*β*	*SE*	95% CI		*t*	*p*	*R*	*R* ^2^	*F*	*p*
				Lower	Upper						
Community feeling	Gender	−0.03	0.03	−0.09	0.02	−1.22	0.223	0.63	0.40	173.45	<0.001
	Only child status	−0.01	0.03	−0.07	0.04	−0.46	0.648				
	Trait depression	−0.63	0.03	−0.68	−0.57	−22.49	<0.001				
Self-compassion	Gender	−0.07	0.02	−0.12	−0.02	−2.88	0.004	0.74	0.55	233.20	<0.001
	Only child status	−0.03	0.02	−0.08	0.02	−1.12	0.262				
	Trait depression	−0.36	0.03	−0.42	−0.30	−11.57	<0.001				
	Community feeling	0.44	0.03	0.38	0.50	14.09	<0.001				
Subjective well-being	Gender	−0.01	0.03	−0.06	0.04	−0.53	0.595	0.72	0.52	168.51	<0.001
	Only child status	0.02	0.03	−0.03	0.07	0.82	0.415				
	Trait depression	−0.51	0.03	−0.58	−0.44	−14.65	<0.001				
	Community feeling	0.14	0.04	0.07	0.22	4.02	<0.001				
	Self-compassion	0.15	0.04	0.07	0.22	3.97	<0.001				

**Table 3:** Mediating Effects of Each Sub-Model and the Overall Model.

Path	Ratio (%)	Effect	*SE*	Bias-Corrected 95% CI	
				Lower	Upper
Total indirect effect	26.09	−0.18	0.03	−0.24	−0.13
Path 1: TD→CF→SWB	13.04	−0.09	0.03	−0.14	−0.04
Path 2: TD→SC→SWB	7.25	−0.05	0.01	−0.08	−0.03
Path 3: TD→CF→SC→SWB	5.80	−0.04	0.01	−0.06	−0.02

Notes: TD = Trait depression; CF = Community feeling; SC = Self-compassion; SWB = Subjective well-being.

## Data Availability

The data presented in this study will be made available upon reasonable request.
